# BPRF: Blockchain-based privacy-preserving reputation framework for participatory sensing systems

**DOI:** 10.1371/journal.pone.0225688

**Published:** 2019-12-05

**Authors:** Hyo Jin Jo, Wonsuk Choi

**Affiliations:** 1 Department of Software, Hallym University, Chuncheon-si, Gangwon-do, Republic of Korea; 2 Graduate School of Information Security, Korea University, Seoul, Republic of Korea; University of Maryland, UNITED STATES

## Abstract

Participatory sensing is gaining popularity as a method for collecting and sharing information from distributed local environments using sensor-rich mobile devices. There are a number of participatory sensing applications currently in wide use, such as location-based service applications (e.g., Waze navigation). Usually, these participatory applications collect tremendous amounts of sensing data containing personal information, including user identity and current location. Due to the high sensitivity of this information, participatory sensing applications need a privacy-preserving mechanism, such as anonymity, to secure and protect personal user data. However, using anonymous identifiers for sensing sources proves difficult when evaluating sensing data trustworthiness. From this perspective, a successful participatory sensing application must be designed to consider two challenges: (1) user privacy and (2) data trustworthiness. To date, a number of privacy-preserving reputation techniques have been proposed to satisfy both of these issues, but the protocols contain several critical drawbacks or are impractical in terms of implementation. In particular, there is no work that can transparently manage user reputation values while also tracing anonymous identities. In this work, we present a blockchain-based privacy-preserving reputation framework called BPRF to transparently manage user reputation values and provide a transparent tracing process for anonymous identities. The performance evaluation and security analysis show that our solution is both practical and able to satisfy the two requirements for user privacy and data trustworthiness.

## Introduction

**Fig 1:**
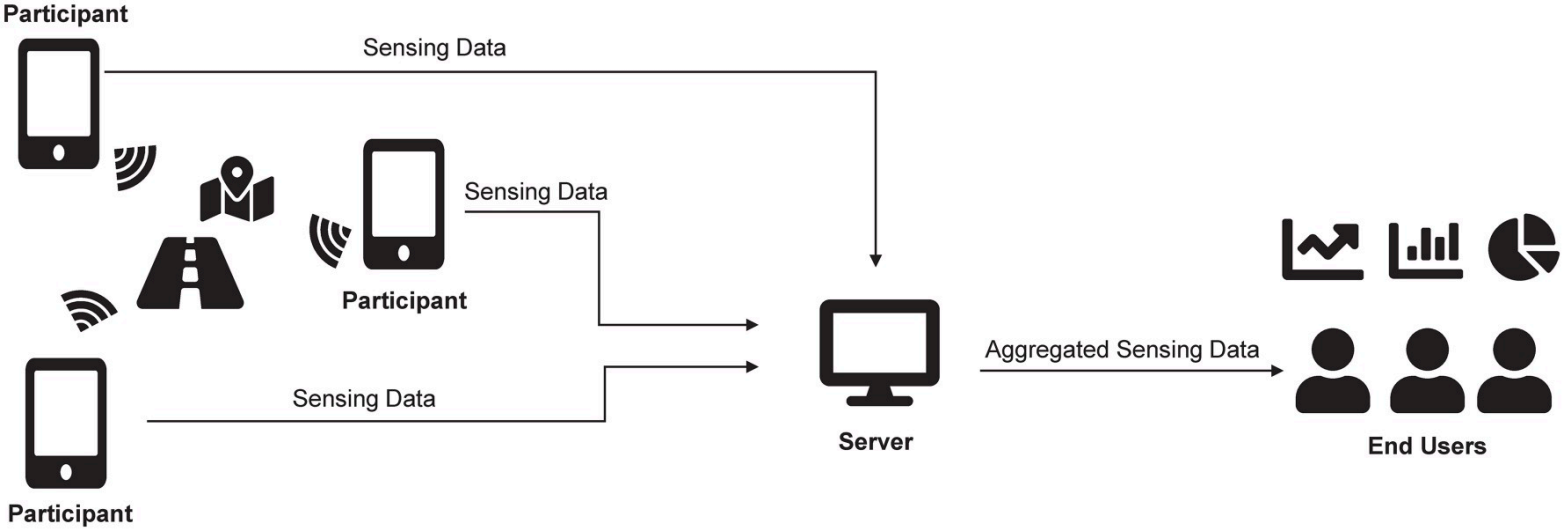
Participatory sensing system.

With the ever-rapid development of mobile communication technologies in recent years, sensor-rich mobile devices such as smartphones and tablet PCs have become common extensions of everyday life. By leveraging this mobile environment, participatory sensing systems have attracted a great deal of attention [1]. In participatory sensing, distributed users with mobile devices collect data from surrounding environments and upload them to an application server via mobile communication technologies (e.g., 4G/5G or Wi-Fi). The application server aggregates uploaded data from multiple users, then identifies meaningful information by analyzing the aggregated data. The analyzed results are provided to users who have registered for the relevant participatory sensing services. Fig 1 shows the basic architecture of a participatory sensing system.

Participatory sensing architecture can be applied to a variety of applications like location-based services [2] (e.g., Waze navigation) and item-locating services [3] (e.g., TrackR).

In general, applications for participatory sensing require a high level of participation from users in order to acquire information that is useful and accurate. However, private user information could potentially be leaked as a direct result of the high level of participation. For example, in Waze, users share information about surrounding road conditions in real time (e.g., accidents and congestion) via location information (i.e., GPS coordinates) to contribute to Waze’s services. Thus, it is clear that the Waze server can track drivers who contribute information about road conditions to the server by using transmitted location information.

To encourage users to participate in these participatory sensing applications, private user information such as identity or location information must be protected from unauthorized access. As one example, anonymity could be guaranteed for users who have registered in a participatory sensing system.

However, protection methods for private user information like anonymity could impair data trustworthiness of a participatory system because malicious users may abuse the anonymity and input fake information, damaging the reliability of these services. To ensure data trustworthiness, the participatory sensing system requires a function that is used to evaluate misleading, i.e. fake, information that has been uploaded by malicious users. Keeping track of user reputation based on user behavior history could be one way to evaluate the quality and reliability of sensing information.

From this perspective, a successful participatory sensing application must satisfy two requirements: (1) user privacy and (2) data trustworthiness. However, meeting these two requirements simultaneously is not a simple task. User privacy is often supplied by eliminating links between successive user actions (e.g., successive sensing information from one user), but such links are usually required to evaluate data trustworthiness. For example, in a pseudonym-based participatory sensing system, a user may have a set of pseudonyms and switch between them each session to make their contributions difficult to link. Owing to such erratic behavior, a participatory sensing system is virtually unable to trace a single user by analyzing contributed data in the system. (In this paper, “trace” means finding out the real identity of an anonymous user). On the other hand, using a pseudonym makes influencing or manipulating user reputation more difficult for any user because user behavior is usually monitored via a unique identifier, not multiple identities (i.e., pseudonyms). In general, the management of reputation values is used to ascertain whether or not the data is trustworthy. Still, even though data trustworthiness is not entirely dependent on user reputation values, there is a general correlation between a high probability of reported data being trustworthy and high user reputation values.

### Contributions

In this paper, we present a blockchain-based privacy-preserving reputation framework, BPRF, that takes a practical approach and considers both user privacy and data trustworthiness. BPRF is composed of a potentially large set of participating users, a blockchain, and two types of servers (application servers (AS) and a tracing server (TS)) that are assumed to not collude.

In BPRF, a group signature algorithm and a partially blind signature algorithm are used to protect private user information. AS is responsible for registering users who want to join participatory sensing applications offered by itself. During registration, all users are assigned to the default reputation level by AS and obtain the corresponding group member keys. After registration, a user can transmit sensing information with a group signature value to AS’s participatory application. Successful contribution to AS’s application offers the contributing user a reward token that is signed by AS via a partially blind signature algorithm. The tokens issued by AS are then transmitted to a blockchain (i.e., smart contract) charged with managing user reputation values. During each instance of reputation update, AS assigns each user to a new reputation level that has been determined by each user’s reputation value. The number of reputation levels depends on AS’s privacy policy. If there is a dispute regarding fake information potentially sent by a malicious user, TS is able to trace the action back to the user by collaborating with the application server. In summary, this paper makes the following contributions:

- Reputation values of users are designed to be transparently managed by a blockchain, so that all entities can audit the history of their own reputation values. Auditing reputation values is useful for dealing with unauthorized editing, such as cases involving an abnormal increases or decreases in reputation values by a reputation system.
- BPRF adopts a group signature algorithm to provide anonymity to users. Each group represents a specific reputation level and not a specific user. In addition, BPRF is designed to record trace events in the blockchain to prevent unauthorized tracing of anonymous users. Thus, whenever a group signature value is traced to identify which user transmitted it, information related to the trace event is logged as a blockchain transaction.
- By inserting recognizable information, such as that reputation level, to reward tokens issued by AS, the tokens become nontransferable to other users at different reputation levels.

The remainder of this paper is structured as follows: In the second section, we introduce related works and the building blocks of our proposed framework. Motivation and architecture of the proposed framework are discussed in the third section. We then present our privacy-preserving reputation framework (BPRF) in the fourth section. Performance evaluation and security analysis are described in the fifth section. In the sixth section, we discuss the reputation management policy, the problem of collusion, the blockchain overhead and the response way to malicious users of the proposed framework, respectively. The paper is concluded in the seventh section.

## Related works and building blocks

### Related works

To provide privacy-preserving reputation techniques, some studies have been proposed in [4–12], but these approaches have several drawbacks.

In [4] and [5], an application server that manages user reputation values can also trace user behavior. Thus, these studies are not secure against an honest-but-curious server model, in which a server is assumed to always follow protocol, but may try to glean unauthorized (or private) information from protocol logs beyond the permitted parameters. In [6], malicious users cannot be traced or revoked from anonymous groups because the system provides untraceable anonymity. In other words, no one is able to uncover the real identity of a malicious user. Likewise, the study in [7] is unable to detect or trace abnormal use of a reputation value. For example, if a malicious user with a low reputation value were to abuse the high reputation value made available in partnership with colluding users, the abnormal behavior would neither be detected nor flagged by the application server.

While the studies of [4–7] cannot address privacy-preserving reputation thoroughly, the works of [8–12] are also insufficient to be applied to diverse participatory sensing application environments. Zhai *et al*. proposed an anonymous reputation system based on the existence of many pseudonyms generated by collaborations over multiple servers rather than a single server [8]. However, this approach is inefficient in time-sensitive participatory sensing application functions, such as Waze’s time-sensitive information sharing system, due to the length of time required to generate one pseudonym —around 2,000 seconds when there are 100,000 participating users. This is an unacceptable delay given the nature of time-sensitive applications. That is, if this work were applied to Waze, a user would have to wait 2,000 seconds to receive a new pseudonym in order to transmit a new traffic event back to the Waze server. The work [8] provides a parallel operation option to reduce the time it takes to generate a new pseudonym. However, since there is a trade-off between the parallel operation option and privacy, the parallel operation option is still limited. Likewise, the studies in [9–12] are only applied to online purchasing systems because users in these studies are allowed to share some information only if they have a previous purchase history. Finally, to the best of our knowledge, it is important to note that there are no works that transparently manage user reputation values. Managing the reputation management process and the tracing process transparently is pertinent for improving the reliability of privacy-preserving reputation management.

### Building blocks

#### Group signature

In group signature algorithms, a set of users is assigned to one group. A user included in a group then signs a message anonymously without revealing his/her identity by using the group identity [13]. In general, group signature algorithms should provide controllable anonymity to cope with potential abuse of anonymity features [14, 15]. For example, a group manager who managing parameters of group signature algorithms can trace a signer through its own specific group signature value if there is a dispute related to the signer. The functions required by the controllable group signature are described as follows.

- GS.Initialize_By_Server(k_1_)→GPK, GTK, GIK, GLK: With input k_1_ (a pre-defined security parameter), this function outputs GPK, GTK, GIK and GLK, which are a group public key, a group tracing key, a group issuing key, and a group link key, respectively.
- GS.Initialize_By_User(k_2_) →UPK, USK: With input k_2_ (a pre-defined security parameter) this function outputs UPK and USK, which are user’s public key and private key, respectively.
- GS.Join(UserJoin(GPK, ID, USK),Issue(GPK, GIK, ID, UPK)) → GMK: Two algorithms, UserJoin(GPK, ID, USK) (run by a registered user) and Issue(GPK, GIK, ID, UPK) (run by a group member key issuer), interact to output a group member key GMK that is only stored and managed on the user side. (ID is the identity of a registered user).
- GS.Sig $(\text{GPK},\text{GMK},\text{m})\to \sigma_{\text{GMK}}^{\text{GS}}\left(\text{m}\right)$: With input GPK, GMK, and the message m, this function outputs a group signature $\sigma_{\text{GMK}}^{\text{GS}}\left(\text{m}\right)$
- GS.Ver $(\text{GPK},\sigma_{\text{GMK}}^{\text{GS}}\left(\text{m}\right),\text{m})\to 1$ or 0: On input GPK, $\sigma_{\text{GMK}}^{\text{GS}}\left(\text{m}\right)$, and m, this function outputs 1 (valid) or 0 (invalid).
- GS.Trace $(\text{GPK},\text{GTK},\sigma_{\text{GMK}}^{\text{GS}}\left(\text{m}\right),\text{m})\to \text{UPK}$, *τ*: With input GPK, GTK, *σ*_GMK_(m), and m, this function outputs the signer’s public key (UPK) and a proof *τ*.
- GS.Judge $(\text{GPK},\text{UPK},\sigma_{\text{GMK}}^{\text{GS}}\left(\text{m}\right),\text{m},\tau )\to 1$ or 0: With input GPK, UPK, $\sigma_{\text{GMK}}^{\text{GS}}\left(\text{m}\right)$, m and *τ*, this function outputs 1 (meaning that the owner of UPK generates $\sigma_{\text{GMK}}^{\text{GS}}\left(\text{m}\right)$) or 0.
- GS.Link $(\text{GPK},\text{GLK},\sum \sigma_{\text{GMK}}^{\text{GS}}\left(\text{m}\right),\sum \text{m})\to 1$ or 0: With input GPK, GLK, $\sum \sigma_{\text{GMK}}^{\text{GS}}\left(\text{m}\right)$, and ∑m, this function outputs 1 if there are more than two signatures generated by one user and related to the same message. Otherwise, it outputs 0. ($\sum \sigma_{\text{GMK}}^{\text{GS}}\left(\text{m}\right)$ and ∑m are a set of group signature values and a set of messages, respectively).
- GS.Revoke(GPK, GIK, Revo_List) → GPK^*new*^, Revo_Info: With input GPK, GIK and a revocation list (Revo_List), this function outputs a new group public key (GPK^*new*^) and revocation information (Revo_Info).
- GS.Update(GPK, GPK^*new*^, GMK, Revo_Info) → GMK^*new*^: On input GPK, GPK^*new*^, GMK and Revo_Info, this function outputs a new group member key (GMK^*new*^) if U is not indicated by Revo_Info.

The functions and terms defined in this section have been used to design the proposed framework. BPRF can adopt any kind of group signature algorithms providing above described fucntions. For example, the group signature of [14] can be applied to BPRF.

#### Partially blind signature

The work [16] introduces the concept of “restrictive blind signature”, through which verifying parties can derive some identifying information for authentication. ABE et al. alternatively proposed a method called “partially blind signature”, which allows a signer to include arbitrary public data in a blind signature [17]. The functions described next are basic features provided by a partially blind signature algorithm:

- PB.Initialize(k_3_) → PPK, PSK: With input k_3_ (a pre-defined security parameter), this function outputs a public key (PPK) and the corresponding signing key (PSK) for a partially blind signature.
- PB.Info(common_information) → Info: With input common_information, this function outputs Info.
- PB.Blind(PPK,rand,m) → blind_m_: With input PPK, a random value (rand), and a message m (e.g., an identity), this function outputs a blinded message blind_m_.
- PB.Sig(PPK,PSK,blind_m_,Info) → $\sigma_{\text{PSK}}^{\text{PB}}({\text{blind}}_{\text{m}},\text{Info})$: With input PPK, PSK, info and blind_m_, this function outputs a signature, $\sigma_{\text{PSK}}^{\text{PB}}\left({\text{blind}}_{\text{m}},\text{Info}\right)$, on Info and blind_m_.
- $\text{PB.Unblind}(\text{rand},\sigma_{\text{PSK}}^{\text{PB}}\left({\text{blind}}_{\text{m}},\text{Info}\right))\to \sigma_{\text{PSK}}^{\text{PB}}\left(\text{m},\text{Info}\right)$: With input rand and $\sigma_{\text{PB}}^{\text{PB}}\left({\text{blind}}_{\text{m}},\text{Info}\right)$, this function outputs a signature, $\sigma_{\text{PSK}}^{\text{PB}}\left(\text{m},\text{Info}\right)$, on m and Info.
- $\text{PB.Ver}(\text{PPK},\sigma_{\text{PSK}}^{\text{PB}}\left(\text{m}\right),\text{m})\to 1$ or 0: With input PPK, $\sigma_{\text{PSK}}^{\text{PB}}\left(\text{m}\right)$ and m, this function outputs 1 (valid) or 0 (invalid).

The functions and terms defined in this section have been used to design the proposed framework.

BPRF can adopt any kind of partially blind signature algorithms providing above described functions. For example, the partially blind signature of [17] can be applied to BPRF.

#### Blockchain

A blockchain is a distributed ledger introduced by the Bitcoin protocol in 2008 [18] that is transparently managed by a peer-to-peer network. The blockchain as a secure and decentralized platform has been widely used for applications that need transparent and immutable data management without a centralized entity.

In ethereum [19], smart contracts are defined in a high-level, JavaScript-like language called Solidity (https://solidity.readthedocs.io/en/v0.5.11/) A smart contract that has been formalized in source code can be executed on ethereum nodes without any downtime, centralization, or third-party interference. In general, smart contracts are used to implement useful applications provided by a blockchain. For example, a blockchain with smart contracts can provide a fundraising service that automatically refunds contributions unless a certain amount is raised within a given time frame. In addition to ethereum, other blockchain systems such as Hyperledger [20] and EOS [21] also define their own systems for smart contracts.

## Motivation and architecture

This section presents the motivating scenario and architecture of the proposed framework, BPRF.

### Motivation

**Fig 2:**
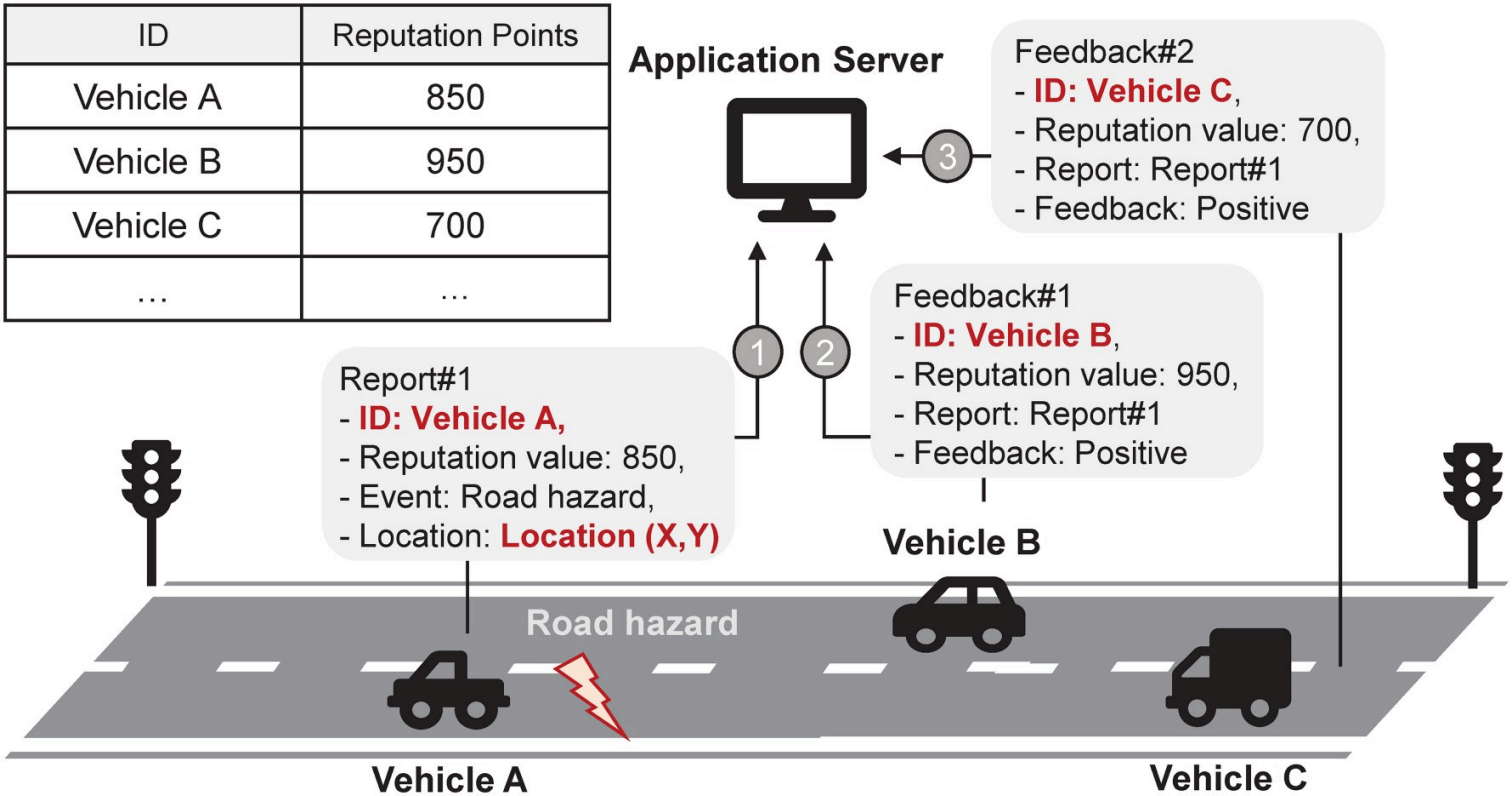
Event sharing system using real identities.

**Fig 3:**
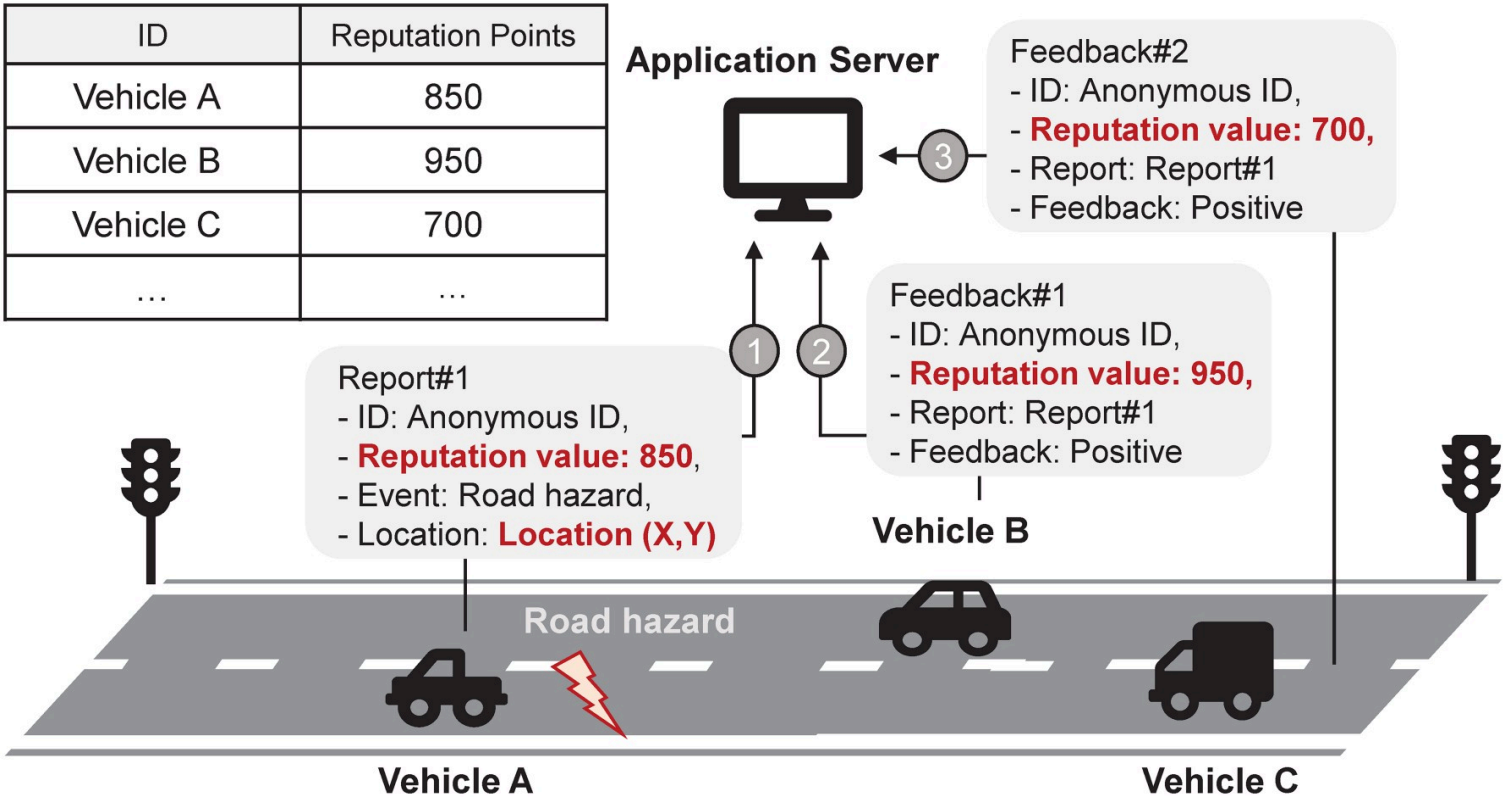
Event sharing system using anonymous identities.

BPRF is motivated by a participatory sensing services like Waze. Figs 2 and 3 show the motivating application examined for this study, which is also an application that could be supported by this proposed framework.

In Fig 2, vehicles A, B, and C transmit reports on nearby road conditions, such as car accidents or construction site updates to AS. Based on the reports, AS posts road events. Other vehicles are allowed to give positive or negative feedback on the posted events when they pass through the areas reportedly affected by the posted events. In general, all vehicles transmitting reports or feedback must do so via a unique, individual identifier specifically for report/feedback message handling processes, such as message authentication or identification of fake information. These unique identifiers are also used to evaluate the reputation of users who have contributed to the participatory sensing system.

However, using unique identifiers means that AS can also monitor and track the location of any vehicle participating in the application. Even when a user does not directly transmit reports about surrounding road conditions to AS, location information of the vehicle can still be leaked to AS if the vehicle transmits feedback of any kind about the reported messages. This unavoidable leakage of private information negatively impacts the popularity and public perception of participatory sensing applications.

Even though all vehicles use pseudonyms as shown in Fig 3, it is still possible to trace some vehicles by linking together a path of reputation values included in reports or feedback messages. For example, the path of vehicle A could be linked by its own reputation value of 850.

### Architecture of BPRF

**Fig 4:**
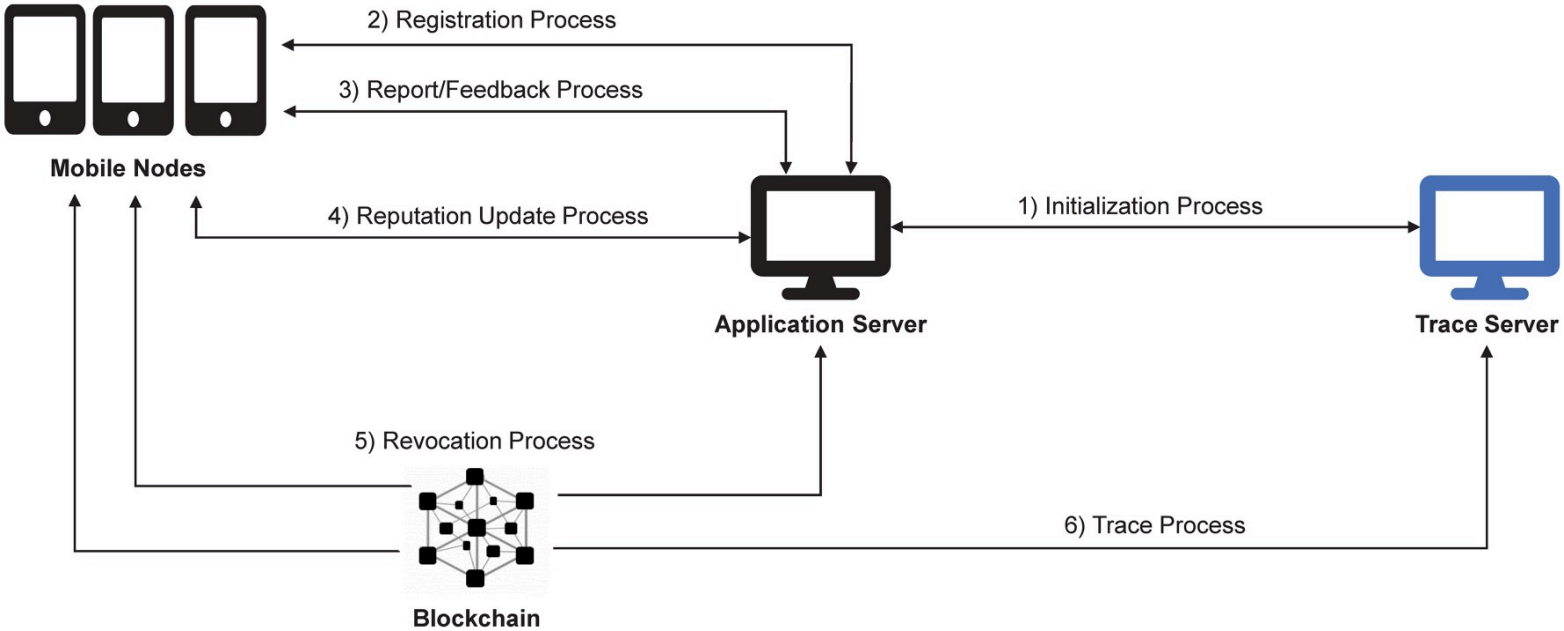
The architecture of BPRF.

The proposed BPRF framework consists of a potentially large number of users that represent a user (**U**), a small number of application servers (AS), a tracing server (TS), and a blockchain. In practice, while each application server has its own applications that are independent from those provided by other application servers, a user can sign up for multiple applications if they wish to subscribe to a variety of services. BPRF allows users to anonymously upload reports or feedback messages to AS. However, if fake reports or feedback messages are detected, a malicious user can be traced through the collaboration of AS and TS. A blockchain is also used to transparently manage reputation values and trace user information. Fig 4 shows the architecture of BPRF.

#### Adversary model and assumptions

While an honest entity in BPRF (a server or a user) follows the BPRF protocol, there are still dishonest users or servers that do not follow the specified BPRF protocol.

On the server side (AS or TS), we consider a honest-but-curious model in which the server is assumed to always follow the protocol, but may try to learn information from the protocol transcript beyond what is intended to be shared [22]. For example, if there is no evidence about an attack attempting to acquire private user information, the honest-but-curious server might use private user information that has been obtained by unauthorized user monitoring without the user’s approval. In addition, the server might try to tamper with reputation values of specific users by assigning a unique reputation value to each, such that the server tracks them by linking together the tampered reputation values. However, some functions such as user registration and the key-issuing process are considered to be honestly performed, as these functions are needed for AS and TS to provide customers with their own services.

On the user side, a malicious user might want to gain access to sensitive information of a specific user by analyzing the user’s reputation value, report messages, or feedback messages. The malicious user can transmit fake sensing reports or fake feedback messages to AS to tamper with the reputation values of other users or itself (e.g., by unfairly increasing or decreasing reputation values). Moreover, multiple malicious users may also collude to track sensitive information of other users, transmit fake messages, or tamper with reputation values.

We assume that each entity has its own public key and the corresponding private key that has been certified by the certified authority (CA). Users and applications are assumed to be connected by anonymous communication channels (e.g., Tor [23], or traffic analysis resistant networks like Dissent [24] and Vuvuzela [25]). Users could also be connected to application servers through a virtual private network (VPN) for efficient communication. We assume that AS and TS do not collude, and that the majority of users are assumed to behave with integrity.

#### Requirements

The requirements of BPRF can be classified into three categories as follows:

Conditional Privacy: User contributions to a participatory system should not disclose any private information, therefore effectively ensuring anonymity and unlinkability. Additionally, traceability is provided to identify misbehaving or otherwise troublesome users. In particular, tracing processes should be audited publicly by all entities.

- Anonymity: We define three types of anonymity: weak, semi-strong, and strong. If weak anonymity is achieved, only third parties are restricted from private user information. Weak anonymity allows one server, such as an application server or a tracing server, to trace anonymous users. Anonymity is semi-strong when anonymous users are only traced if more than two servers collaborate. To clarify, if semi-strong anonymity is achieved, anonymous users cannot be traced by one server as well as a third party. Lastly, strong anonymity is achieved when there are absolutely no entities that can trace anonymous users.
- Unlinkability: No entities should be able to link together user’s report/feedback messages if these messages are valid. Reputation values should also not be used to link an entity to a particular event or events.
- Transparent traceability: If anonymous users misbehave, they should be traced. In addition, tracing processes should be also managed transparently by allowing all entities to audit trace events so that a honest-but-curious server does not attempt to perform a tracing process without valid reasons.

Reputation management: Reputation of users should be accurately managed with transparency, and be updated periodically.

- Correctness: Increase the reputation of users who have contributed to the system in a reliable manner, and decrease the reputation of malicious users via a penalty system. For example, an application server should be able to check whether multiple feedback messages have been transmitted on the same subject, so that reputation does not increase unfairly.
- Transparency: Reputation values should be managed transparently so that all entities can audit their own reputation.
- Update: Reputation values should be updated periodically.

Practicality: In order to apply a privacy-preserving reputation system in real life, system design should be efficient and practical.

## Design of BPRF

### Overview

BPRF is composed of six processes: 1) initialization, 2) registration, 3) report/feedback, 4) reputation update, 5) revocation, and 6) tracing. The notations used in BPRF are explained in Table 1.

**Table 1:** Notations.

Notations	Explanations
TS	A tracing server
AS	An application server
U_*x*_	A user *x*
ID_*x*_	The identity of a user *x*
${\text{blind}}_{{\text{ID}}_{x}}$	The blinded-identity of a user *x*
Time_Info	The time information of AS
UPK_*x*_	The public key of a user *x*
USK_*x*_	The signing key of a user *x*
PPK_AS_	AS’s public key used for a partially blind signature
PSK_AS_	AS’s signing key used for a partially blind signature
$\sigma_{{\text{PSK}}_{\text{AS}}}^{\text{PB}}\left(\text{m}\right)$	The blind signature value of message *m* signed by PSK_AS_
N_RL_	The number of pre-defined reputation levels
RL_*i*_	A reputation level *i*
${\text{GPK}}_{{\text{RL}}_{i}}$	The group public key for RL_*i*_
${\text{GTK}}_{{\text{RL}}_{i}}$	The group tracing key for RL_*i*_
${\text{GIK}}_{{\text{RL}}_{i}}$	The group issuing key for RL_*i*_
${\text{GLK}}_{{\text{RL}}_{i}}$	The group link key for RL_*i*_
GMK_*x*_	The group member key of a user *x*
$\sigma_{{\text{GMK}}_{x}}^{\text{GS}}\left(\text{m}\right)$	The group signature value on message *m* signed by GMK_*x*_
${\text{Revo\_List}}_{{\text{RL}}_{i}}$	The revocation list including all revoked users for each reputation level RL_*i*_
${\text{Revo\_Info}}_{{\text{RL}}_{i}}$	The information of users whose privileges have been revoked from RL_*i*_
Token_Type	The type of tokens: 1) Token_Report, 2) Token_Feedback, 3) Token_Trace
Reward_Token	A reward token is one of two types, i.e., Token_Report or Token_Feedback
Penalty_Token	A penalty token is one type, i.e., Token_Trace

During initialization, TS selects a group signature algorithm that can separately produce and manage a group tracing key, a group issuing key, and a group link key respectively used to trace real identities of malicious users, issue a group member key for every user, and link group signatures. After initializing parameters for the selected group signature algorithm, TS generates a set of group public keys (GPK), group tracing keys (GTK), group issuing keys (GIK), and group link keys (GLK). TS and AS then authenticate each other. If authentication is successful, AS receives all GPKs, GIKs, and GLKs from TS. Every tuple of GPK, GIK, and GLK is mapped to a specific reputation group representing a specific reputation level i (RL_*i*_). For example, if AS has three reputation levels from low, medium, and high, each tuple of GPK, GIK, and GLK is assigned to all three reputation levels.

During registration, a user U_*x*_ registers with AS for the subscription services provided by AS. The user is then assigned to a default reputation group representing a reputation level and generates a corresponding group member key (GMK_*x*_) through collaboration with AS.

In the report/feedback process, a registered user can post reports (e.g., an accident) or feedback of any kind on reports posted by other users along with their own blinded real identities that have been produced by mixing random values with their real identities. Before posting the report or feedback, messages are signed by a group signature algorithm BPRF adopts. If the reports or feedback are successfully verified, each user who has contributed to AS’s application receives a blinded reward token from AS. Each blinded reward token is issued by applying a blind signature algorithm on a blinded real identity (${\text{blind}}_{{\text{ID}}_{x}}$). The users who received blinded reward tokens acquire the tokens (Reward_Token) by eliminating random values, that were used for generation of the blinded real identity, from the blinded reward tokens.

During reputation update, each user that has received the Reward_Tokens, transmits all tokens to a blockchain, i.e., the AS smart contract, which implements the reputation management policy for AS. The smart contract updates reputation values for all users according to the reputation management policy of AS after validation of reward tokens. Through updated reputation values stored in the blockchain, AS can update reputation levels of users. AS then updates public parameters for reputation levels (i.e., reputation groups) to consider membership changes, such as a group member being added or removed. The updated information is also transmitted to all users. The duration of the reputation update period differs from application to application, ranging from a few minutes to a few hours, or even days.

If there is a user that has been assigned to a new reputation level (that is, a new group) after the reputation update, this user is removed from the previous reputation level during revocation.

**Fig 5:**
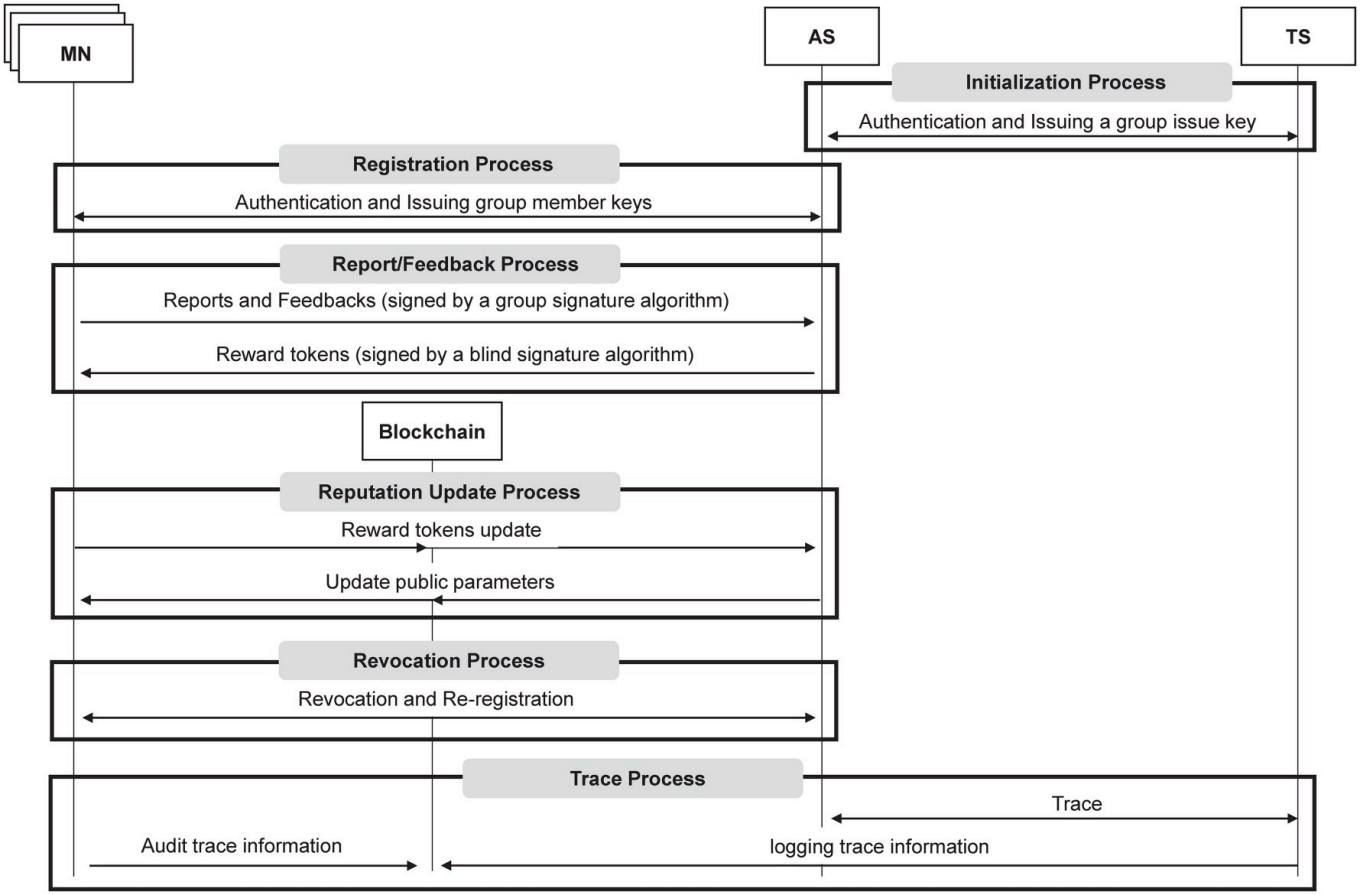
The overview of BPRF.

Whenever AS detects a fake message that could negatively impact the reliability of AS’s services, tracing for the message in question is performed. In this process, the real identity of a fake message sender is traced by collaboration between AS and TS, and then the reputation value of the fake message sender decreases according to the AS reputation management policy. Fig 5 shows the overview of BPRF.

### Initialization

TS selects a group signature algorithm that can separately produce and manage a group tracing key, a group issuing key, and a group link key. AS and TS authenticate each other using a public key infrastructure (PKI). AS sets the number of reputation levels (N_RL_), which is pre-defined in its own reputation policy. AS requests TS to generate N_RL_ tuples of GPK, GTK, GIK, and GLK for its own services. TS then generates the N_RL_ tuples by using GS.Initialize_By_Server(k_1_), where k_1_ is a pre-defined security parameter. While GTKs are stored in TS, GIKs and GLKs are transmitted to AS through a secure channel, like the transport layer security (TLS) channel. GPKs are then publicly announced. In addition, user (U_*x*_) produces a pair of public keys (UPK_*x*_) and a private key (USK_*x*_) through GS.Initialize_By_Server(k_2_), where k_2_ is a pre-defined security parameter.

In order to award a blinded token to users, AS initializes a public key (PPK_AS_) and a signing key (PSK_AS_) via PB.Initialize(k_3_) after selecting a partially blind signature algorithm. (k_3_ is a pre-defined security parameter).

### Registration

During registration, U_*x*_ and AS authenticate each other using PKI. AS then assigns the user to a default reputation level (e.g., a medium reputation level) that has been determined by its own reputation policy.

The user and AS then perform registration. In other words, $\text{UserJoin}({\text{GPK}}_{{\text{RL}}_{\text{i}}},{\text{ID}}_{x},{\text{USK}}_{x})$, and $\text{Issue}({\text{GPK}}_{{\text{RL}}_{i}},{\text{GIK}}_{{\text{RL}}_{i}},{\text{ID}}_{x},{\text{UPK}}_{x})$ are interactively performed by U_*x*_ and AS. ID_*x*_ is the identity of U_*x*_, and ${\text{GPK}}_{{\text{RL}}_{i}}$ and ${\text{GIK}}_{{\text{RL}}_{i}}$ are, respectively, the group public key and group issuing key for the reputation level RL_*i*_ in which U_*x*_ is involved. If registration is successful, U_*x*_ obtains GMK_*x*_, and AS stores a pair of ID_*x*_ and UPK_*x*_ in its own storage.

### Report and feedback

When a user wants to send an alert about an event that could assist other users, it can upload this event to AS by transmitting a report. The uploaded report consists of three parts: 1) an event message (event), 2) a blinded identity (${\text{blind}}_{{\text{ID}}_{x}}$), and 3) a group signature value.

The ${\text{blind}}_{{\text{ID}}_{x}}$ of U_*x*_ is generated by PB.Blind(PPK_AS_, rand, ID_*x*_) after selecting random value (rand), which is used to hide ID_*x*_ from AS. The group signature value, $\sigma_{{\text{GMK}}_{x}}^{\text{GS}}(\text{event}∥{\text{blind}}_{{\text{ID}}_{x}})$, are produced by GS.Sig (${\text{GPK}}_{{\text{RL}}_{i}}$, GMK_*i*_, $\text{event}∥{\text{blind}}_{{\text{ID}}_{x}}$), where ∥ is concatenation.

When the uploaded report is transmitted to AS, $\sigma_{{\text{GMK}}_{x}}^{\text{GS}}(\text{event}∥{\text{blind}}_{{\text{ID}}_{x}})$ is verified through $\text{GS.Ver}({\text{GPK}}_{{\text{RL}}_{i}},\sigma_{{\text{GMK}}_{x}}^{\text{GS}}(\text{event}∥{\text{blind}}_{{\text{ID}}_{x}}),\text{event}∥{\text{blind}}_{{\text{ID}}_{x}})$. Since ${\text{GPK}}_{{\text{RL}}_{i}}$ represents a specific reputation level, AS can determine whether this report should be posted or not through its own posting rules. For example, AS has a rule allowing reports to be posted only when the report is verified by a group public key representing a reputation level higher than medium level.

If a report uploaded by U_*x*_ is successfully posted, the user receives a blinded reward token from AS. The blinded token, $\sigma_{{\text{PSK}}_{\text{AS}}}^{\text{PB}}({\text{blind}}_{{\text{ID}}_{x}},\text{Info})$, is produced by function PB.sig (PPK_AS_, PSK_AS_, ${\text{blind}}_{{\text{ID}}_{x}}$, Info). Function PB.Info (Time_Info ∥RL_*i*_∥ Token_Report) is then used to generate Info.

The user can subsequently retrieve the Reward_Token, $\sigma_{{\text{PSK}}_{\text{AS}}}^{\text{PB}}({\text{ID}}_{x},\text{Info})$, from their blinded token through the function PB.Unblind (rand, $\sigma_{{\text{PSK}}_{\text{AS}}}^{\text{PB}}\left({\text{blind}}_{{\text{ID}}_{x}},\text{Info}\right)$) which eliminates random value (rand), which was used to produce the blinded identity.

Likewise, all users can upload feedback on a report to update the situation, or offer a positive or negative personal opinion. The feedback consists of three parts: 1) feedback on a posted report (feedback), 2) a blind identity (${\text{blind}}_{{\text{ID}}_{x}}$), and 3) a group signature value ($\sigma_{{\text{GMK}}_{x}}^{\text{GS}}(\text{feedback}∥{\text{blind}}_{{\text{ID}}_{x}})$). The group signature value is also verified by the corresponding ${\text{GPK}}_{{\text{RL}}_{i}}$. The feedback can be used to evaluate the posted report. It should be noted that designing a report evaluation algorithm using feedback messages such as EigenTrust [26] is out of the scope of this paper.

Users who contribute feedback also receive blinded reward tokens, $\sigma_{{\text{PSK}}_{\text{AS}}}^{\text{PB}}({\text{blind}}_{{\text{ID}}_{x}},\text{Info})$, from AS. Function PB.Info (Time_Info ∥RL_*i*_∥ Token_Feedback) is used to generate Info. The reward token, $\sigma_{{\text{PSK}}_{\text{AS}}}^{\text{PB}}({\text{ID}}_{x},\text{Info})$, can also be retrieved from the blinded tokens through function PB.Unblind (rand, $\sigma_{{\text{PSK}}_{\text{AS}}}\left({\text{blind}}_{{\text{ID}}_{x}},\text{Info}\right)$).

Additionally, AS is required to check duplicate report messages or feedback messages to prevent its own service from becoming biased due to duplicate messages. By using function GS.Link (${\text{GPK}}_{{\text{RL}}_{i}}$, ${\text{GLK}}_{{\text{RL}}_{i}}$, group signatures, messages), AS in BPRF can detect duplicate reports and feedback messages representing the same event or feedback content that have also been generated by one user. Thus, users that have generated duplicate report messages or feedback messages receive Reward_Token only once from AS.

**Algorithm 1**: Reputation Update

**Input**: a token (Reward_token or Penalty_token)

             // e.g., $\sigma_{{\text{PSK}}_{\text{AS}}}^{\text{PB}}({\text{ID}}_{x},\text{Info})$, PPK_AS_, (ID_*x*_, Info)

**Output**: 0 or 1

**1**

 /* This is a (key, value) structure like Solidity mapping   */

**2** Map (string → int) ID_Map   // ID → reputation_value

**3 ** Map (int → int) Token_Map   // Token → a flag

**4**

 /* This is a verification step using PB.Ver(…)   */

**5** **if** *Token verification using PB.Ver(…)* **then**

**6**

  /* This is a parsing step using Info that is the result of PB.Info(…)   */

**7**  String ID = ID        // from the token

**8**  int time_info = Time_Info   // from the token

**9**  int RL = RL_*i*_        // from the token

**10**  int token_type = Token_Type // from the token

**11**

  /* This is a step to check whether or not time_info is valid and the token is unused          */

**12**  **if** *(time_info is not expired) and (the token is unused)* **then**

**13**   int reputation = ID_Map(ID)    // ID’s reputation_value

**14**   Token_Map(the token) = 1  //Setting flags to check for token usage

**15**

 /* This is one reputation update step   */

**16**   **if** *Reputation is in the range of RL* **then**

**17**    **if** *token_type ⩵ Token_Report* **then**

**18**     ID_Map(ID) = reputation + report_point

**19**     return 1

**20**    **else if** *token_type ⩵ Token_Feedback* **then**

**21**     ID_Map(ID) = reputation + feedback_point

**22**     return 1

**23**    **else if** *token_type ⩵ Token_Trace* **then**

**24**     ID_Map(ID) = reputation—penalty_point

**25**     return 1

**26**    **else**

**27**     return 0

**28**   **else**

**29**    return 0

**30**  **else**

**31**   return 0

**32** **else**

**33**  return 0

### Reputation update

Users can upload the reward tokens they earned to AS’s smart contract that manages reputation update when they wish to use them. According to the pre-defined reward policy of AS, the smart contract can increase reputation values for any user who has transmitted reward tokens. Algorithm 1 is the pseudo code of AS’s smart contract for reputation update. However, if a user interacts with AS’s smart contract for the reputation update directly after receiving a reward token for a submitted report or feedback, there is a possibility that the user’s identity will become associated with the event message sent. To handle this issue, we have introduced a random delay-based token transmission process. In this random delay-based token transmission process, the user must wait a random amount of time to send his/her own reward tokens. A random delay is determined by random number *r* chosen between 0 and *K*, where *K* is a system parameter for this random delay. When the number of reputation update requests that can be checked through algorithm 1 transactions reaches random number *r*, the user sends his/her own reward tokens accumulated while waiting for *r* reputation update requests to AS’s smart contract for reputation update. Thus, all tokens a user earned can be processed as a batch. If *K* is large enough, the possibility of information leakage is minimized.

For each predetermined reputation update period, AS updates reputation levels of all users by reading reputation values stored in the permanent storage of its own smart contract.

### Revocation

If there are users who have been assigned to new reputation levels after the reputation update process, AS generates revocation lists (e.g., $\text{Revo}\_{\text{List}}_{{\text{RL}}_{i}}$) that include all users whose reputation levels have changed. In the revocation process, AS performs GS.Revoke $\left({\text{GPK}}_{{\text{RL}}_{i}},{\text{GTK}}_{{\text{RL}}_{i}},\text{Revo}\_{\text{List}}_{{\text{RL}}_{i}}\right)$ to generate ${\text{GPK}}_{{\text{RL}}_{i}}^{new}$ and $\text{Revoke}\_{\text{Info}}_{{\text{RL}}_{i}}$. AS then broadcasts ${\text{GPK}}_{{\text{RL}}_{i}}^{new}$ and $\text{Revoke}\_{\text{Info}}_{{\text{RL}}_{i}}$ to all users involved in the reputation level *i* (RL_*i*_) to trigger the update of group member keys. For example, a user (U_*x*_) can update its own group member key though GS.Update (${\text{GPK}}_{{\text{RL}}_{i}}$, ${\text{GPK}}_{{\text{RL}}_{i}}^{new},$ GMK_*x*_, $\text{Revoke}\_{\text{Info}}_{{\text{RL}}_{i}}$) that outputs ${\text{GMK}}_{x}^{new}$.

In order to compromise with the time-sensitive nature of the report and feedback process while performing GS.Update (${\text{GPK}}_{{\text{RL}}_{i}}$, ${\text{GPK}}_{{\text{RL}}_{i}}^{new},$ GMK_*x*_, $\text{Revoke}\_{\text{Info}}_{{\text{RL}}_{i}}$), BPRF sets a grace period for the amount of time that a user continues to retain or use its own group parameters after these parameters expire. The grace period as a system parameter is set longer than the time required to perform GS.Update (${\text{GPK}}_{{\text{RL}}_{i}}$, ${\text{GPK}}_{{\text{RL}}_{i}}^{new},$ GMK_*x*_, $\text{Revoke}\_{\text{Info}}_{{\text{RL}}_{i}}$). If a user (U_*x*_) has been revoked from reputation level *i* and the grace period is over, the user can no longer use the previous GMK_*x*_ because it is not verifiable by ${\text{GPK}}_{{\text{RL}}_{i}}^{new}$ anymore.

### Tracing

The tracing process is triggered when AS detects a malicious report. The detection of a malicious report can be done through predefined abnormal conditions, such as a flood of negative feedback on a single posted report or a flood of reports that are inconsistent with other posted reports. The definition of abnormal conditions varies from application to application. If AS detects a malicious report, AS sends the report to TS. Then, TS executes the tracing function, GS.trace (${\text{GPK}}_{{\text{RL}}_{i}},$ ${\text{GTK}}_{{\text{RL}}_{i}},$ $\sigma_{{\text{GMK}}_{x}}^{\text{GS}}(\text{event}∥{\text{blind}}_{{\text{ID}}_{x}})$, $\text{event}∥{\text{blind}}_{{\text{ID}}_{x}}$), which outputs UPK_*x*_ of a malicious user and the corresponding *τ*, which can be used to prove that $\sigma_{{\text{GMK}}_{x}}^{\text{GS}}(\text{event}∥{\text{blind}}_{{\text{ID}}_{x}})$ is related to UPK_*x*_. After finding UPK_*x*_ from the malicious report, TS sends $\sigma_{{\text{GMK}}_{x}}^{\text{GS}}(\text{event}∥{\text{blind}}_{{\text{ID}}_{x}})$, UPK_*x*_, and *τ* to AS’s smart contract that is managing trace events. This contract validates whether $\sigma_{{\text{GMK}}_{x}}^{\text{GS}}(\text{event}∥{\text{blind}}_{{\text{ID}}_{x}})$ is correlated with UPK_*x*_ by using *τ*. The information in this tracing process, $\sigma_{{\text{GMK}}_{x}}^{\text{GS}}(\text{event}∥{\text{blind}}_{{\text{ID}}_{x}})$, UPK_*x*_, and *τ*, is stored as transactions of AS’s smart contract for trace management.

**Algorithm 2**: Tracing

**Input**: Trace information

          // i.e., ${\text{GPK}}_{{\text{RL}}_{i}}$, UPK_*x*_, $\sigma_{{\text{GMK}}_{x}}^{\text{GS}}\left(m\right)$, m, *τ*

**Output**: 0 or 1

 /* This is a (key, value) structure similar to Solidity mapping   */

**1** Map (byte[] → int) UPK_Map       //UPK_*x*_ → counter

**2**

  /* This is a validation step using GS.Judge(…)       */

**3** **if** *Validation of proof(τ) using GS.Judge(…)* **then**

**4**  int counter = UPK_Map(UPK_*x*_)

**5**  UPK_Map(UPK_*x*_) = counter + 1

**6**  return 1

**7** **else**

**8**  return 0

Algorithm 2 is pseudo code for AS’s smart contract for trace management. Whenever the algorithm 2 is called up by AS, the counter value of a traced user increases by 1 if trace proof *τ* has been validated successfully. If the counter value manged by algorithm 2 is greater than the threshold, a penalty token (Penalty_Token), i.e., $\sigma_{{\text{PSK}}_{\text{AS}}}^{\text{PB}}\left({\text{ID}}_{x},\text{Info}\right)$, is issued by AS, in which Info is generated by PB.Info (Time_Info ∥RL_*i*_∥ Token_Trace). Then, Penalty_Token is transmitted to algorithm 1 to decrease reputation value as per Penalty_Token.

Through this tracing process, the reputation level of a user who has sent a malicious report to AS can be reevaluated.

## Evaluation and analysis

In this section, we evaluate the performance of and analyze BPRF to verify whether or not it satisfies the requirements set out in the third section of this paper.

### Evaluation

For evaluation, we assume that BPRF adopts a group signature and a partially blind signature, as proposed in [14] and [17], respectively. These algorithms were chosen exclusively to measure the performance of BPRF, but the selection of eligible BPRF algorithms is not limited to these two alone. In other words, BPRF can adopt any kind of group signature or partially blind signature algorithm that has its functions outlined in the building blocks section.

We do not evaluate the performance of algorithm 1 or algorithm 2 because the performance of these is dependent on blockchain types (e.g., Ethereum, Hyperledger, EOS). However, we expect that these algorithms can still be implemented practically. For example, in Ethereum, the algorithms can be implemented via a Solidity-based pairing library [27].

#### Implementation

For evaluation of BPRF, we implemented two basic cryptographic operations, exponentiation and pairing [28], which are required for the execution of [15] and [17] schemes. Lightweight operations such as the cryptographic hash function are ignored because they are negligible compared to the basic operations we implemented. We used the Java Pairing-Based Cryptography Library (jPBC) library [29] for implementation of exponentiation and pairing operations. During implementation, d159 parameters provided by the jPBC library were used. (A type D curve is defined over a given field *F*_*q*_ and has an order *h* × *r* where *r* is a prime and *h* is a small constant. Over the field $F_{q}^{6}$, its order is a multiple of *r*^2^. https://crypto.stanford.edu/pbc/manual/ch08s06.html) The implemented code can be found in the following link (https://github.com/emsecurity/BPRF).

Our implementation was tested on two devices: an Intel Processor i7-8700 @ 3.20 GHz and a Xiaomi POCO F1 smartphone. After each operation was executed 1000 times from each device, the average execution time was obtained. Table 2 shows the average execution time for each operation.

**Table 2:** Implementation of basic crypto operations.

	i7-8700 (at 3.20 GHz)	Xiaomi POCO F1
Pairing	14.24 *ms*	368 *ms*
Exponentiation	1.78 *ms*	13.5 *ms*

#### Evaluation of cryptographic functions

Table 3 illustrates evaluation results for GS.Sig(), GS.Ver(), GS.Trace(), GS.Judge(), GS.Link(), GS.Revoke(), GS.Update(), and PB.Info(), PB.Blind(), PB.Sig(), PB.Unblind(), PB.Ver() when BPRF adopts the algorithms [14] and [17]. Since initialization and registration are only performed once per user in BPRF, we did not evaluate the functions required in these processes, which are: GS.Initialize_By_server(), GS.Initialize_By_Client(), GS.Join(), and PB.Initialize().

**Table 3:** Evaluation (*r*: Number of revoked users, *neg*: Negligible time, −: The function is not executed on the target device). †: This can be implemented faster by using multi-exponentiation and Java Native Interface (JNI) [30].

	Server (e.g., AS, TS) (i7-8700 at 3.20GHz)	Client (e.g., U_*x*_) (Xiaomi POCO F1)
GS.Sig()	−	135 *ms*
GS.Ver()	33.82 *ms*	−
GS.Trace()	8.9 *ms*	−
GS.Judge()	35.6 *ms*	−
GS.Link()	28.48 *ms*	−
GS.Revoke()	8.9 *ms*	−
GS.Update()	−	135,000 ms† (If *r* = 10,000)
PB.Info()	*neg*	−
PB.Blind()	−	27 *ms*
PB.Sig()	3.56 *ms*	−
PB.Unblind()	−	*neg*
PB.Ver()	1.78 *ms*	13.5 *ms*

A number of basic operations required for executing each function of [14] and [17] are described as follows.

- GS.Sig(): The signing process requires no pairing computation and only 10 exponentiations.
- GS.Ver(): The verifying process requires 11 exponentiations and one pairing computation.
- GS.Trace(): The tracing process requires 5 exponentiations.
- GS.Judge(): The judging process requires 4 exponentiations and two pairing computations.
- GS.Link(): The linking algorithm requires 2 pairing computations.
- GS.Revoke(): The revocation process requires requires 5 exponentiations.
- GS.Update(): If the number of revoked users is *r*, the revocation process requires 3 ⋅ *r* exponentiations.
- PB.Info(): The setting process of common information does not require any basic operations.
- PB.Blind(): The blinding process requires 2 exponentiations.
- PB.Sig(): The signing process requires 2 exponentiations.
- PB.Unblind(): The unblinding process does not require any basic operations. It simply requires arithmetic operations with small numbers like square operations that are also negligible compared to basic operations.
- PB.Ver(): The verifying process requires 1 exponentiation.

In addition, the performance of BPRF is only compared with the work [8], as it provides semi-strong anonymity like our framework. In this comparison, a preparation step (i.e., update of a pseudo-identity or update of a group member key), report generation and verification steps, feedback generation and verification steps, and token generation and verification steps are compared, as shown in Table 4.

**Table 4:** Overhead comparison between BPRF and the work of [8]. |*R*|: Number of members in a ring signature group ^†^: The result was measured in AMAZON EC2 c4.8xlarge virtual machines when five servers are used for the preparation step. ^‡^: ELGamal.Sig() and ELGamal.Ver() require 1 exponentiation and 3 exponentiations, respectively. ^§^: Linkable.Ring.Sig() and Linkable.Ring.Vig() of [31] require 7 exponentiations and 4 exponentiations, respectively.

		Zhai et al. [8]	BPRF
Preparation		1,000 s^†^ (Performed every time per message, N = 100,000)	135 s (Performed periodically, N = 100,000)
Report	Generation (by a user)	13.5 ms (= ELGamal.Sig()^‡^)	135 ms (= PB.Blind() + GS.Sig())
	Verification (by a server)	40.5 ms (= ELGamal.Ver()^‡^)	33.82 ms (= GS.Ver())
Feedback	Generation (by a user)	94.5 ms (= Linkable.Ring.Sig()^§^)	135 ms (= PB.Blind() + GS.Sig())
	Verification (by a server)	7.12 ms × |*R*| (= Linkable.Ring.Ver()^§^)	33.82 ms (= GS.Ver())
Reward Token	Generation (by a server)	-	3.56 ms (PB.Sig() + PB.Unblind())
	Verification (by a user)	-	13.5 ms (= PB.Ver())

Through this comparison, we found that BPRF is more efficient than [8], which performs an additional preparation step (i.e., update of a pseudo-identity) per message to provide unlinkability between successive reports or feedback uploaded by a user.

### Security analysis

BPRF is analyzed in terms of the following requirements.

#### Conditional privacy

- Anonymity: When a user (U_*x*_) wants to send a report message or feedback message to AS, it signs the message using its group member key that represents a reputation level (i.e., a reputation group). Since a group signature value does not correspond to any one particular user, AS cannot know precisely which user transmitted the message. TS is also unable to trace a group signature value without help from AS because the group signature value is only transmitted to AS, and the transmission channel between U_*x*_ and AS is assumed to be protected by TLS. The corresponding group signature value is transmitted to TS from AS only when there is a dispute related to a report message. After that, the signature value is traced by TS. Thus, BPRF provides semi-strong anonymity according to the categories outlined in third section.
- Unlinkability: BPRF is assumed to use an anonymous network channel, like Tor. This means that AS cannot link U_*x*_ using a network identifier (e.g., IP addresses). Additionally, a group signature value represents not a specific reputation value, but a reputation level. Since each reputation level is set to include a large number of users, AS cannot link activities from a user (U_*x*_) using group signature values.
- Transparent traceability: The presence of a malicious report message can be traced via collaboration between AS and TS.
  In addition, the traced information is also included in the blockchain’s immutable storage by means of algorithm 2. Thus, a honest-but-curious AS is not able to send a tracing request to TS without a valid tracing reason because TS records all tracing requests in the blockchain to provide transparent audits.

#### Reputation management

- Correctness: BPRF is designed to protect the reputation values of all users from attacks that influence, alter, or manipulate reputation values. By adopting the blockchain’s transparent and immutable storage, no one including AS or TS can intentionally modify user reputation values.
  The only way to update reputation values is to send reward tokens (by users) or trace tokens (by TS) to the smart contract, which manages reputation values. When AS issues reward tokens to users who have contributed to its own service, a check for other identical report messages or feedback messages by any one user is performed in order to protect reputation values from self-increasing attempts, in which a user deliberately generates duplicate messages to improve its own reputation value.
  In addition, reward tokens are designed to include contributing user reputation information (i.e., reputation levels), meaning that they cannot be transferred to other users belonging to different reputation levels. For example, a user assigned to the highest reputation level cannot transfer any of its own reward tokens to a user assigned to the lowest reputation level. This feature prevents an attacker with the low reputation value from collecting reward tokens from other accomplice belonging to the highest reputation level. Lastly, as described in algorithm 1, time information and double spending of tokens are also checked.
- Transparency: Reputation values of all users are managed by a smart contract described in algorithm 1, and these are stored in the blockchain. In addition, the information stored by algorithm 2 can be used to audit trace events. Thus, all users can audit their own reputation values managed in the blockchain storage.
- Update: The reputation values for all users are refreshed every predefined update interval. For example, users who have contributed to AS’s service are eligible to receive a Reward_Token. The reputation values of users that have adversely affected the service of AS by sharing fake messages are decreased via a Penalty_Token.

#### Practicality

BPRF is based on a group signature algorithm, a partially blind signature algorithm, and a blockchain that can be practically implemented with the jPBC library and Ethereum’s Solidity. Implementation and evaluation results in Tables 2 and 3 illustrate the practicality of BPRF.

Table 5 compares BPRF and related works in terms of conditional privacy and reputation management requirements.

**Table 5:** Comparison (W: Weak, SS: Semi-Strong, S: Strong).

		[4]	[5]	[6]	[7]	[8]	BPRF
Conditional Privacy	Anonymity	W	W	S	S	SS	SS
	Unlinkability	X	X	O	O	O	O
	Transparent Traceability	X	X	X	X	X	O
Reputation Management	Correctness	O	O	O	X	O	O
	Transparency	X	X	O	X	X	O
	Update	O	O	O	O	O	O

## Discussion

### Reputation management policy

**Fig 6:**
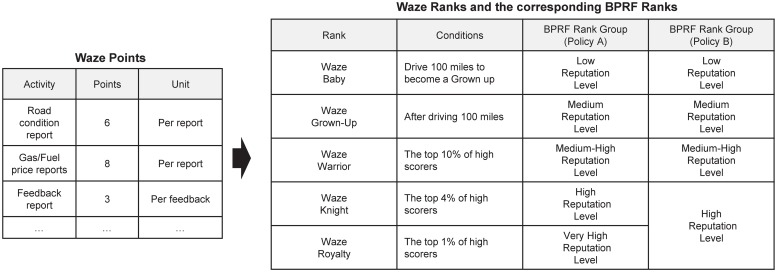
An example of reputation management policy.

Although every participatory application has its own reputation management policy, it is possible to apply BPRF to a number of such applications. In this section, we give one example (i.e., Waze) to show how BPRF can be applied to real-life applications. As shown in Fig 6, Waze has a policy for user-earned points and ranking levels. If a Waze user sends a report about road conditions, the user is awarded six points, and the top one percent of high scorers in a given region are classified as Waze Royalty.

In this example, we present two BPRF policies: policy A and policy B. In policy A, five Waze ranking levels and five levels of BPRF directly correspond to each other as shown in Fig 6. However, since the number of users belonging to Waze Royalty (the top one percent of high scorers) is small, the behavior of users belonging to this very high reputation level could be easily linked to specific areas. Therefore, in policy B, Waze Knight and Waze Royalty levels are combined to form the highest reputation level. As such, BPRF policies can be defined and customized for participatory application environments.

### Collusion between AS and TS

We assume that AS and TS do not collude with each other to infringe on user privacy. However, if AS and TS were to collude to maliciously trace a user without logging the corresponding trace event in a blockchain, BPRF would not provide user privacy.

To cope with this potential collusion problem, BPRF can be modified as follows. First, a pseudo-identity generation process is added between BPRF initialization and registration, and then registration for BPRF itself is changed. For this process, a set of pseudonym servers is needed to issue pseudo-identities to users. First, a user (U_*x*_) sends its own identity (ID_*x*_) to the set of servers. Then, the servers encrypt ID_*x*_ to generate the pseudo-identity (PID_*x*_) via a threshold public-key encryption [32, 33], whereupon, if performed successfully, PID_*x*_ is securely transmitted to U_*x*_. After PID_*x*_ passes through this additional process, U_*x*_ performs a modified registration process. In this modified process, UserJoin(${\text{GPK}}_{{\text{RL}}_{\text{i}}}$, PID_*x*_, USK_*x*_) and Issue(${\text{GPK}}_{{\text{RL}}_{i}}$, ${\text{GIK}}_{{\text{RL}}_{i}}$, PID_*x*_, UPK_*x*_) are interactively performed by U_*x*_ and AS instead of UserJoin(${\text{GPK}}_{{\text{RL}}_{\text{i}}}$, ID_*x*_, USK_*x*_) and Issue(${\text{GPK}}_{{\text{RL}}_{i}}$, ${\text{GIK}}_{{\text{RL}}_{i}}$, ID_*x*_, UPK_*x*_) as described in the original registration process.

Thus, in the modified version of BPRF, AS and TS cannot trace a user even if they collude because the user has been registered in AS by using its own pseudo-identity. To trace a malicious user in the modified version of BPRF, TS stores the corresponding trace event including PID_*x*_ in a blockchain and then the pseudonym servers cooperatively decrypt PID_*x*_ stored in the corresponding trace event. Since a threshold public-key encryption is designed to decrypt a ciphertext only if at least *t* pseudonym servers cooperate, a system parameter *t* is set to handle collusion problems among pseudonym servers or failures of pseudonym servers.

In addition, since the pseudo-identity generation process by the pseudonym servers is performed only once before registration, the pseudonym servers do not incur processing overhead like the work of [8]. In other words, a user does not need to generate a pseudo-identity from the pseudonym servers each time, as the process only need be performed at the time of registration, and BPRF is designed to use a group member key to send reports or feedback.

If the work of [8] were designed to allow a user to use one pseudo-identity, the process of issuing a pseudo-identity in the study could be streamlined to one time. However, in [8], if a user used only one pseudo-identity, any other users could link the behavior of that specific pseudo-identity. In other words, the use of one pseudo-identity does not provide unlinkability in [8]. On the other hand, in the modified version of BPRF, even if a user uses one pseudo-identity, the behavior of a specific pseudo-identity is linked only when AS and TS collude. This means that the modified version of BPRF only allows for the colluding servers to link the behavior of a specific pseudo-identity, not any and all users. This linkability issue among colluding servers will be addressed in future work.

### Blockchain overhead

The use of a blockchain could affect performance of BPRF. However, the report/feedback generation processes can be performed without a blockchain, as shown in the Fig 4. In other words, processes associated with blockchain—reputation updates, revocation, and traces are executed periodically rather than each time. Thus, they do not significantly affect the time-sensitive report/feedback process for information sharing.

In addition, a blockchain such as Ethereum requires the user or a server to pay a fee (i.e., Ethereum gas payments) for the execution of the smart contract codes, such as algorithms 1 and 2. As such, the cost of using a blockchain could be a burden on both the user and the server. To handle this issue, users and servers can have blockchain transactions processed in batches by using a scalability solution like Plasma [34] that allows for decentralized applications to move transactions off of a root blockchain. BPRF could also consider using EOS or Hyperledger, which allows for free and fast execution of smart contract codes.

### Response to malicious users

In a participatory sensing system, abnormal behavior (e.g., fake reports) of a malicious user can be detected by a “flood of negative feedback”. However, the system should also consider a cautious adversary who deliberately generates duplicate messages that only slightly deviate from what may be considered normal behavior to improve its own reputation value.

For example, in Waze, a malicious user can upload multiple reports with slightly varied GPS information to unfairly earn more reward tokens. To prevent malicious users from potentially generating multiple reports under different identities, the Waze server divides a region into several report domains. This subsequently enables the server to set a policy that does not allow multiple reports of the same type in one domain to curb malicious user behavior. For example, in Waze, there are 10 types of reports: Traffic, Police, Crash, Hazard, Map chat, Map issue, Place, Roadside help, Camera, and Closure reports. Implementation of this policy could manifest into actions such as not giving reward tokens to delayed report submissions of the same type, or reducing reputation points accumulated by reward tokens received for delayed report submissions.

It should be noted that response methods vary from application to application. In line with this, participatory sensing systems should be able to handle the behavior of malicious users in their own way.

## Conclusion

A privacy-preserving reputation system is needed for participatory sensing applications in order to protect user privacy and to evaluate the reputation of contributors who relay information about their environments to the system. However, building a privacy-preserving reputation system that guarantees the two requirements of user privacy and data trustworthiness is not a simple task. It seems natural that such a participatory sensing system dealing with these two requirements would be highly demanded, as it would simultaneously provide user privacy and data trustworthiness. As such, in this paper, we proposed a blockchain-based privacy-preserving reputation framework (BPRF) for participatory sensing applications. In BPRF, user reputation values can be publicly audited through a blockchain-based reputation system. In addition, our framework includes an auditable tracing process, in which trace events of anonymous users are logged in the immutable storage of the blockchain. Thus, under BPRF, a honest-but-curious server is unable to trace an anonymous user without any valid grounds for doing so. This paper includes our analysis of the proposed framework and evaluation results of its performance. For future research, we aim to develop a smart contract for BPRF that would be distributed over a blockchain network, such as Ehtereum, EOS, or Hyperledger.

## References

[1] Burke J, Estrin D, Hansen M, Parker A, Ramanathan N, Reddy S, et al. Participatory sensing. In: Workshop on World-Sensor-Web (WSW’06): Mobile Device Centric Sensor Networks and Applications; 2006. p. 117–134.

[2] Méndez D, Pérez AJ, Labrador MA, Marrón JJ. P-Sense: A participatory sensing system for air pollution monitoring and control. In: 2011 IEEE International Conference on Pervasive Computing and Communications Workshops (PERCOM Workshops); 2011. p. 344–347.

[3] Agadakos I, Polakis J, Portokalidis G. Techu: Open and Privacy-Preserving Crowdsourced GPS for the Masses. In: Proceedings of the 15th Annual International Conference on Mobile Systems, Applications, and Services. MobiSys’17. New York, NY, USA: ACM; 2017. p. 475–487. Available from: http://doi.acm.org/10.1145/3081333.3081345.

[4] Huang KL, Kanhere SS, Hu W. A privacy-preserving reputation system for participatory sensing. In: 37th Annual IEEE Conference on Local Computer Networks; 2012. p. 10–18.

[5] Garms L, Martin K, Ng SL. Reputation Schemes for Pervasive Social Networks with Anonymity. In: Proceedings of the fifteenth International Conference on Privacy, Security and Trust (PST 2017). IEEE; 2017.

[6] Kokoschka A, Petrlic R, Sorge C. A Reputation System Supporting Unlinkable, Yet Authorized Expert Ratings. In: Proceedings of the 30th Annual ACM Symposium on Applied Computing. SAC’15. New York, NY, USA: ACM; 2015. p. 2320–2327. Available from: http://doi.acm.org/10.1145/2695664.2695892.

[7] Wang XO, Cheng W, Mohapatra P, Abdelzaher T. ARTSense: Anonymous reputation and trust in participatory sensing. In: 2013 Proceedings IEEE INFOCOM; 2013. p. 2517–2525.

[8] Zhai E, Wolinsky DI, Chen R, Syta E, Teng C, Ford B. AnonRep: Towards Tracking-Resistant Anonymous Reputation. In: 13th USENIX Symposium on Networked Systems Design and Implementation (NSDI 16). Santa Clara, CA: USENIX Association; 2016. p. 583–596. Available from: https://www.usenix.org/conference/nsdi16/technical-sessions/presentation/zhai.

[9] Blömer J, Juhnke J, Kolb C. In: Böhme R, Okamoto T, editors. Anonymous and Publicly Linkable Reputation Systems. Berlin, Heidelberg: Springer Berlin Heidelberg; 2015 p. 478–488. Available from: 10.1007/978-3-662-47854-7_29.

[10] Busom N, Petrlic R, Sebé F, Sorge C, Valls M. A privacy-preserving reputation system with user rewards. Journal of Network and Computer Applications. 2017;80(Supplement C):58–66. 10.1016/j.jnca.2016.12.023

[11] Azad MA, Bag S, Hao F. PrivBox: Verifiable decentralized reputation system for online marketplaces. Future Generation Computer Systems. 2018;89:44–57. 10.1016/j.future.2018.05.069

[12] Bag S, Azad MA, Hao F. A privacy-aware decentralized and personalized reputation system. Computers & Security. 2018;77:514–530. 10.1016/j.cose.2018.05.005

[13] Chaum D, van Heyst E. Group Signatures. In: Davies DW, editor. Advances in Cryptology—EUROCRYPT’91. Berlin, Heidelberg: Springer Berlin Heidelberg; 1991. p. 257–265.

[14] Hwang JY, Lee S, Chung BH, Cho HS, Nyang D. Group signatures with controllable linkability for dynamic membership. Information Sciences. 2013;222:761–778. 10.1016/j.ins.2012.07.065

[15] Hwang JY, Chen L, Cho HS, Nyang D. Short Dynamic Group Signature Scheme Supporting Controllable Linkability. IEEE Transactions on Information Forensics and Security. 2015;10(6):1109–1124. 10.1109/TIFS.2015.2390497

[16] Brands S. Untraceable Off-line Cash in Wallet with Observers. In: Proceedings of the 13th Annual International Cryptology Conference on Advances in Cryptology. CRYPTO’93. Berlin, Heidelberg: Springer-Verlag; 1994. p. 302–318. Available from: http://dl.acm.org/citation.cfm?id=188105.188172.

[17] Abe M, Fujisaki E. How to date blind signatures In: Kim K, Matsumoto T, editors. Advances in Cryptology—ASIACRYPT’96. Berlin, Heidelberg: Springer Berlin Heidelberg; 1996 p. 244–251.

[18] Nakamoto S. Bitcoin: A Peer-to-Peer Electronic Cash System; 2008. Available from: https://bitcoin.org/bitcoin.pdf.

[19] Wood G. Ethereum: A Secure Decentralised Generalised Transaction Ledger-EIP-150 Revision;. Available from: https://gavwood.com/paper.pdf.

[20] Hyperledger;. Available from: https://www.hyperledger.org/.

[21] EOS.IO Technical White Paper v2;. Available from: https://github.com/EOSIO/Documentation/blob/master/TechnicalWhitePaper.md.

[22] Huang Y, Katz J, Evans D. Quid-Pro-Quo-tocols: Strengthening Semi-honest Protocols with Dual Execution. In: 2012 IEEE Symposium on Security and Privacy; 2012. p. 272–284.

[23] Dingledine R, Mathewson N, Syverson P. Tor: The Second-generation Onion Router. In: Proceedings of the 13th Conference on USENIX Security Symposium—Volume 13. SSYM’04. Berkeley, CA, USA: USENIX Association; 2004. p. 21–21. Available from: http://dl.acm.org/citation.cfm?id=1251375.1251396.

[24] Wolinsky DI, Corrigan-Gibbs H, Ford B, Johnson A. Dissent in Numbers: Making Strong Anonymity Scale. In: Presented as part of the 10th USENIX Symposium on Operating Systems Design and Implementation (OSDI 12). Hollywood, CA: USENIX; 2012. p. 179–182. Available from: https://www.usenix.org/conference/osdi12/technical-sessions/presentation/wolinsky.

[25] van den Hooff J, Lazar D, Zaharia M, Zeldovich N. Vuvuzela: Scalable Private Messaging Resistant to Traffic Analysis. In: Proceedings of the 25th Symposium on Operating Systems Principles. SOSP’15. New York, NY, USA: ACM; 2015. p. 137–152. Available from: http://doi.acm.org/10.1145/2815400.2815417.

[26] Kamvar SD, Schlosser MT, Garcia-Molina H. The Eigentrust Algorithm for Reputation Management in P2P Networks. In: Proceedings of the 12th International Conference on World Wide Web. WWW’03. New York, NY, USA: ACM; 2003. p. 640–651. Available from: http://doi.acm.org/10.1145/775152.775242.

[27] Optimal ate pairing over Barreto-Naehrig curves;. Available from: https://github.com/adjoint-io/pairing.

[28] Menezes A. In: An introduction to pairing-based cryptography; 1991.

[29] The Java Pairing Based Cryptography Library (JPBC);. Available from: http://gas.dia.unisa.it/projects/jpbc/.

[30] Paik JH, Seo SC, Kim Y, Lee HJ, Jung H, Lee DH. An Efficient Implementation of Block Cipher in Android Platform. In: 2011 Fifth FTRA International Conference on Multimedia and Ubiquitous Engineering; 2011. p. 173–176.

[31] Liu JK, Wong DS. Linkable Ring Signatures: Security Models and New Schemes In: Gervasi O, Gavrilova ML, Kumar V, Laganà A, Lee HP, Mun Y, et al, editors. Computational Science and Its Applications—ICCSA 2005. Berlin, Heidelberg: Springer Berlin Heidelberg; 2005 p. 614–623.

[32] Shoup V, Gennaro R. Securing Threshold Cryptosystems against Chosen Ciphertext Attack. Journal of Cryptology. 2002;15(2):75–96. 10.1007/s00145-001-0020-9

[33] Delerablée C, Pointcheval D. Dynamic Threshold Public-Key Encryption In: Wagner D, editor. Advances in Cryptology—CRYPTO 2008. Berlin, Heidelberg: Springer Berlin Heidelberg; 2008 p. 317–334.

[34] Poon J, Buterin V. Plasma: Scalable autonomous smart contracts; 2017. Available from: https://plasma.io/plasma.pdf.

